# A pulmonary artery false aneurysm after right middle lobectomy: a case report

**DOI:** 10.1186/1752-1947-1-70

**Published:** 2007-08-25

**Authors:** Hossam Shaaban, Hemant Sharma, Jagan Rao, Stephen Clark

**Affiliations:** 1 Cardiothoracic Centre, Freeman Hospital, Newcastle upon Tyne, UK; 2 Department of Vascular Surgery, Frenchay Hospital, Bristol, UK

## Abstract

Pulmonary artery false aneurysm is a rare condition, reported to complicate interventional procedures. We report a case of a false aneurysm of the interlobar pulmonary artery following a right middle lobectomy for lung cancer. This is probably the first reported case.

## Background

Pulmonary artery false aneurysm is a rare condition, more common in females and with advancing age. It is related to an increase in pulmonary artery pressure [1] and has been described as a complication of vascular interventional procedures [2]. However, pulmonary artery pseudoaneurysm as a postoperative complication of pulmonary resection is unreported. We report a case of a false aneurysm of the interlobar pulmonary artery following a right middle lobectomy.

## Case presentation

**Figure 1:**
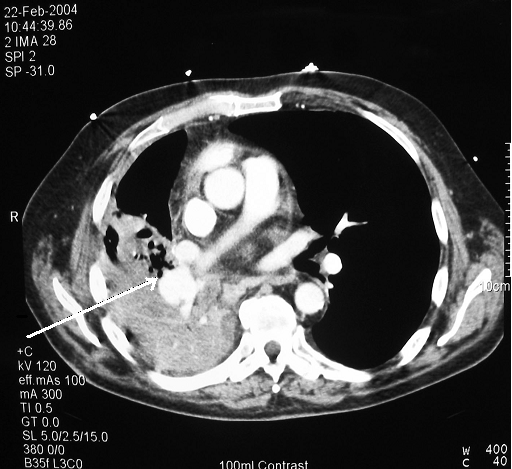
False aneurysm of the right descending interlobar artery (arrow). Note consolidation of the lung parenchyma.

A 69-year-old gentleman underwent a right middle lobectomy for a Stage 1A squamous cell carcinoma of his right middle lobe. The operation involved stapling the pulmonary veins using TX30V stapler and double ligation of the pulmonary artery branches. The bronchus was stapled using a TA30 stapler and the minor fissure was completed with a TCT75 linear cutting stapler. The early post-operative course was unremarkable. The patient however was readmitted five weeks postoperatively with pyrexia and an episode of haemoptysis. Chest X-ray showed haziness in the right mid-zone and the patient was treated for a presumed chest infection. He improved and was discharged. A week later, he was readmitted with continuing haemoptysis, swinging pyrexia and increasing shortness of breath at rest. Bronchoscopy revealed granulation tissue near the middle lobe bronchial stump, which bled profusely on biopsy. Blood appeared to be venous in origin. A clot in the lower lobe bronchus was found together with an inflamed and narrowed lower lobe orifice. CT scan showed a false aneurysm of the right descending interlobar pulmonary artery and complete lower lobe collapse and consolidation (Fig 1). The diagnosis of a bronchovascular fistula complicating a false aneurysm was made and the patient was re-explored with a view to completion right lower lobectomy.

At the second thoracotomy, there were dense and vascular adhesions throughout the pleural space, which was completely obliterated. Mobilisation of the lung was extremely difficult. The entire lung was consolidated and solid. The pulmonary veins were exceedingly friable and attempt to encircle them caused the vessels to tear and result in massive bleeding. In order to maintain vascular control, a pneumonectomy was considered the safest option under the circumstances. Postoperatively, the patient recovery was unremarkable and was discharged home on the eighth postoperative day.

**Figure 2:**
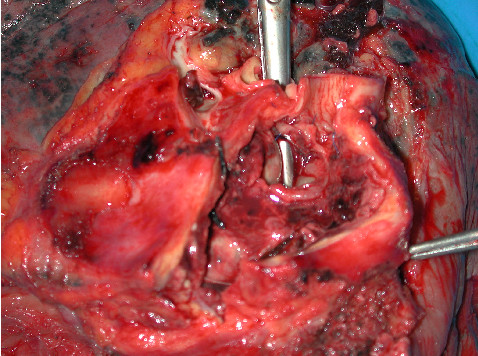
The false aneurysm cavity was incised and retracted open. Dissecting scissors were inserted into the bronchus intermedius. These can be clearly seen through the bronchovascular fistula.

Macroscopic examination of the bilobectomy specimen showed a grossly consolidated lung, distorted by adhesions and pleural thickening. On incision of the pulmonary artery, atheromatous change was noted in the proximal branching of the artery and the false aneurysm cavity was seen communicating with the bronchus intermedius (Fig 2). Microscopic examination of the specimen showed a quite marked atheroma of the pulmonary artery and a false aneurysm with adjacent abscess like inflammatory mass. Lung parenchyma showed an organising pneumonia, oedema and intra-alveolar fibrin.

Examination of the bronchial margin and the lung parenchyma showed no residual carcinoma.

## Discussion

False aneurysms result from rupture of all three structural layers of the arterial wall, usually due to penetrating or blunt trauma. False aneurysm of the pulmonary artery has been described in cases of Behcet's syndrome, neoplasia, pulmonary aspergillosis, septic emboli and chest trauma [3-7]. It can also be iatrogenic, especially after Swan Ganz catheter insertion and balloon inflation [2]. The aetiology in our case can simply be due to inadvertent trauma to the pulmonary artery in the first surgery. However, careful pathological examination of the excised lung from the second surgery strongly suggests infection as a precipitating factor for the development of the aneurysm in an already weakened, atherosclerotic pulmonary artery branch. Moreover, an increase in pulmonary arterial pressure post lobectomy is well documented [8], and could have had a role in the aetiology.

The gold standard for diagnosis used to be pulmonary angiography, but this is now largely superseded by non-invasive CT angiography and 3-D reconstruction. Treatment can be surgical through aneurysmectomy and/or lobectomy or radiological through steel or tungsten coil embolization. In our case, however, owing to a grossly consolidated lung and friable pulmonary vessels, pneumonectomy was elected as the safest option.

## Competing interests

The author(s) declare that they have no competing interests.

## Authors' contributions

HS was involved in collection of data and material used during preparation of the paper. HS was involved in the editing and drafting of the paper. JR was involved in the primary and revision surgery and contributed to the initial draft. SC is the lead clinician involved in the care of the patient and the prime supervisor of the work. All authors read and approved the final manuscript.
